# *MMP-9 *gene variants increase the risk for non-atopic asthma in children

**DOI:** 10.1186/1465-9921-11-23

**Published:** 2010-02-24

**Authors:** Leonardo A Pinto, Martin Depner, Norman Klopp, Thomas Illig, Christian Vogelberg, Erika von Mutius, Michael Kabesch

**Affiliations:** 1 Pontificia Universidade Católica do Rio Grande do Sul, Porto Alegre, Brazil; 2 University Children's Hospital, Ludwig Maximilian's University Munich, Munich, Germany; 3 Helmholtz Zentrum Muenchen, German Research Center for Environmental Health, Germany; 4 University Children's Hospital Dresden, Technical University, Dresden, Germany; 5 Department of Pediatric Pneumology and Neonatology, Hannover Medical School, Hannover, Germany

## Abstract

**Background:**

Atopic and non-atopic wheezing may be caused by different etiologies: while eosinophils are more important in atopic asthmatic wheezers, neutrophils are predominantly found in BAL samples of young children with wheezing. Both neutrophils as well as eosinophils may secrete matrix metalloproteinase 9 (MMP-9). Considering that MMP-9 plays an important role in airway wall thickening and airway inflammation, it may influence the development of obstructive airway phenotypes in children. In the present study we investigated whether genetic variations in *MMP-9 *influence the development of different forms of childhood asthma.

**Methods:**

Genotyping of four HapMap derived tagging SNPs in the *MMP-9 *gene was performed using MALDI-TOF MS in three cross sectional study populations of German children (age 9-11; N = 4,264) phenotyped for asthma and atopic diseases according to ISAAC standard procedures. Effects of single SNPs and haplotypes were studied using SAS 9.1.3 and Haploview.

**Results:**

SNP rs2664538 significantly increased the risk for non-atopic wheezing (OR 2.12, 95%CI 1.40-3.21, p < 0.001) and non-atopic asthma (OR 1.66, 95%CI 1.12-2.46, p = 0.011). Furthermore, the minor allele of rs3918241 may be associated with decreased expiratory flow measurements in non-atopic children. No significant effects on the development of atopy or total serum IgE levels were observed.

**Conclusions:**

Our results have shown that homozygocity for *MMP-9 *variants increase the risk to develop non-atopic forms of asthma and wheezing, which may be explained by a functional role of MMP-9 in airway remodeling. These results suggest that different wheezing disorders in childhood are affected differently by genetic alterations.

## Background

Wheezing affects app. 50% of children up to the age of six, and many but not all of these children develop persistent wheezing or asthma later in life [1]. Diverse wheezing phenotypes can be identified in hindsight based on differences in natural histories, trigger and risk factors [2,3]. Atopic and non-atopic wheezing can easily be discriminated in children by the presence or absence of sensitization to allergens. Atopic and non-atopic asthma show contrasting natural histories and may be caused by different etiologies: while eosinophils are most prevalent in bronchoalveolar lavage (BAL) of older atopic asthmatic wheezers [4], neutrophils are predominantly found in BAL samples of young children with severe wheezing [5].

When severe airway inflammation with the involvement of neutrophilic inflammation is ongoing [6,7], repair processes may contribute significantly to airway remodeling and irreversibility of lung injury. For repair and remodeling, matrix metalloproteinases (MMPs), a family of proteases that degrade components of the extracellular matrix, seem to be of central importance. Neutrophils synthesize MMP-9 and the protease is stored in specific granules from which it is readily released. In circumstances in which neutrophils are abundant, MMP-9 can be secreted, and identifying other cellular sources of MMP-9 may be difficult. In patients with respiratory diseases, macrophages [8], eosinophils [9] mast cells [10], and dendritic cells [11] all may produce MMP-9 under specific stimulation [8,9]. Eosinophils, important in asthma-related airway inflammation may also be a source of MMP9 and MMP9 expression correlate with eosinophil counts in bronchial mucosa [12]. Accordingly, matrix metalloproteinase 9 (MMP-9) acts as a pro-inflammatory molecule perpetuating immune responses [13] but it is also involved in repair processes after tissue injury and may down regulate remodeling during inflammatory reactions. As a consequence of its ambivalent nature, MMP-9 levels in sputum have been directly related to airway inflammation and inversely associated with airway thickening at the same time [7].

Based on the relevance of MMP-9 in both inflammation and airway remodeling [7,14-18], it was hypothesized that genetic variations in the MMP-9 gene could be involved in different forms of wheezing and asthma. A number of polymorphisms have previously been described for the *MMP-9 *gene, including variations in the promoter (rs3918241 = A-1831T) and coding region (rs2664538 = A2659G) resulting in decreased MMP-9 expression and activity [19]. Thus, the association between four haplotype tagging SNPs capturing all essential genetic information of the *MMP-9 *gene locus (rs3918241, rs2664538, rs3918256 and rs3787268) and wheezing phenotypes was studied in three cross sectional population samples of German children.

## Methods

### Population description

Between 1995 and 1996, a cross sectional study was performed in Munich and Dresden as part of the International Study of Asthma and Allergy in Childhood phase II (ISAAC II) to assess the prevalence of asthma and allergies in 5,629 schoolchildren, age 9 to 11 years. All children of German origin with DNA available were included in this analysis (N = 3,099). Additionally, 1,165 fourth graders from Leipzig, phenotyped with a very similar protocol were also analyzed. As described in detail before [20], self-administered questionnaires included the ISAAC core questions on symptoms and diagnoses of asthma, hay fever, and atopic eczema. Informed written consent was obtained from all parents of children included in these studies and all study methods were approved by the local ethics committees.

Children whose parents reported a physician's diagnosis of asthma once, or recurrent, spastic or asthmatic bronchitis more than once were classified as having asthma. Children were categorized as having current wheezing if the parents had reported wheeze in the past 12 months in Munich and Dresden or persistent wheezing in combination with a confirmative answer to the question "had your child ever had wheezing" in Leipzig.

The sensitivity to common aeroallergens (Dresden and Munich: *Dermatophagoides pteronyssinus, Dermatophagoides farinae, Alternaria tenuis*, cat dander, and mixed grass and tree pollen; Leipzig: *Dermatophagoides pteronyssinus*, grass, birch and hazel pollen, cat and dog dander) was assessed by skin prick test (SPT) in all children included in the study (3054 negative/1056 positive/154 missing = 4264). A child was considered atopic if a wheal reaction ≥ 3 mm occurred to one or more allergens after subtraction of the negative control. Atopic asthma was defined as asthma and the concomitant presence of a positive SPT, while non-atopic asthma was defined as asthma in the absence of a positive skin prick test. As controls for atopic or non-atopic asthma children without asthma and without atopy were used. Atopic/non-atopic wheeze was defined accordingly (current wheeze and/without concomitant presence of a positive SPT). Total serum IgE levels were measured using the Imulite System (DPC Biermann, Germany). Specific IgE antibodies (*Sx1 *from Phadia, Germany) against inhalative allergens (local grass pollen, birch pollen, mugwort pollen, *dermatophagoides pteronyssinus*, cat dander, dog dander, *cladosporium herbarum*) were measured in a range between 0.35-100 IU/ml.

In 9-11 yr old children from Munich and Dresden, lung function was measured by MasterScope Version 4.1 (Jäger, Würzburg, Germany). A minimum of two baseline spirograms was performed and the highest of two reproducible measurements of forced expiratory volume in one second (FEV1) was recorded as baseline FEV1[21]. Reproducible measurements of maximum expiratory flows (MEF) at 25, 50 and 75% of vital capacity, MMEF (maximum mid-expiratory flow: the average expiratory flow over the middle half of the forced vital capacity, FVC) were determined.

### SNPs selection and genotyping

The *MMP-9 gene *had previously been screened for polymorphisms [22]. Based on all 11 SNPs genotyped by HapMap[23] with a minor allele frequency (MAF) >0.05 in the European population, linkage disequilibrium (r^2 ^> 0.8) was assessed using the software Haploview [24] and four haplotype tagging SNP capturing the genetic information of all common SNPs at the locus were selected for genotyping. Of note, reference SNP id rs2664538 was recently changed into rs17576, dbSNP but for better comparability with previous publications we continued to refer to rs2664538 in the text.

Genomic DNA was extracted from whole blood by a standard salting out method [25] and pre-amplified as described before[26]. DNA samples were genotyped using matrix-assisted laser desorption/ionization time-of-flight (MALDI-TOF) mass spectrometry (Sequenom Inc., San Diego, California). Additional details on genotyping are available in the online supplement.

Genotyping SNPs in different populations may lead to a recruitment bias. However this may be almost excluded in our study considering the following facts: (1) this is a truly cross-sectional study that included all fourth graders in the respective cities. (2) Admixture was tested between a random sample of 400 children from Munich and 400 samples from Dresden [27]. Considering this data, bias caused by admixture is very unlikely in these population samples. (3) Investigation of the fine structure of European populations with applications to association studies [27] have shown that slight differences observed between these populations cannot explain differences in associations.

### DNA pre-amplification and MALDI-TOF

To minimize the use of genomic DNA in further analyses, a modified primer extension pre-amplification (PEP) or alternatively the GenomiPhi procedure (Ameham Biosciences, Freiburg, Germany) were applied for random DNA pre amplification [28]. PCR assays and associated extension reactions were designed using the SpectroDESIGNER software (Sequenom Inc., San Diego, California). All amplification and extension reaction conditions have been previously described [29] and specific primers are given in table 1. Primer extension products were loaded onto a 384-element chip with a nanoliter pipetting system (SpectroCHIP, SpectroJet, Sequenom) and analyzed by a MassARRAY mass spectrometer (Bruker Daltonik GmbH, Bremen, Germany). The resulting mass spectra were analyzed for peak identification using the SpectroTYPER RT 2.0 software (Sequenom Inc., San Diego, California). For quality control, Hardy-Weinberg calculations were performed to ensure that each marker was within the expected allelic population equilibrium.

**Table 1:** Successful genotyping call rates (Call R in %), minor allele frequencies (MAF), and test for deviation from Hardy-Weinberg Equilibrium (pHWE)

bin	SNP number	Position (to ATG)	Position in the Gene Structure	MAF (HapMap database)	rs Number	LD with tagging SNP	Tagging SNP	MAF (ISAAC sample)	CallR%	test for HWE**
1	1	-1831	Promotor	0.19	rs3918241	1.0	rs3918241	0.15	91.65	NS

	4	2127	Intron 4	0.19	rs2274755	1.0				

	6	3009	Intron 6	0.19	rs2236416	1.0				

	9	5545	Exon 12*	0.19	rs2274756	1.0				

	10	6026	Exon 13*	0.22	rs3918261	0.82				

	11	7773	3'	0.19	rs3918270	1.0				

	2	570	Intron 1	0.40	rs3918249	0.96				

2	5	2659	Exon 6*	0.38	rs2664538	1.0	rs2664538	0.36	94.63	NS

	3	1945	Intron 3	0.46	rs3918253	1.0				

3	7	3394	Intron 7	0.46	rs3918256	1.0	rs3918256	0.44	95.97	NS

4	8	4165	Intron 8	0.18	rs3787268	1.0	rs3787268	0.22	91.84	NS

* coding SNPs, ** p value for comparison between observed genotyping frequencies and frequencies estimated by Hardy-Weinberg equilibrium were not significant (NS).

### Bioinformatics

Transcription factor (TF) binding analyses were performed using *FASTSNP *and *Alibaba TF search*. Phylogenetic comparisons with mouse and dog sequences were performed using the Vista Genome Browser http://pipeline.lbl.gov/cgi-bin/gateway2. Conserved non-coding sequence (CNS) was defined as a sequence longer than 100 bp and more than 70% conserved with mouse or rat.

### Statistical Analysis

SNPs were tested for deviation from Hardy-Weinberg equilibrium (HWE) using chi-square tests, with expected frequencies derived from allele frequencies. Association between SNPs and dichotomous outcomes were tested using chi-square in a recessive model. Additionally odds ratio and 95% confidence intervals are given. We calculated associations for all genetic models in the first population sample (Dresden). While additive and dominant models showed similar results, the recessive model showed the most consistent effects of SNPs in *MMP-9 *in the Dresden dataset and was thus applied to all datasets. Therefore, we present only the recessive model, based on robustness and consistency in different populations.

Lung function parameters were calculated as percentage of reference values. To test for differences in lung function parameters between genotypes t-tests in a recessive model were used. All tests were two-sided and the differences were considered significant with p < 0.05. To correct for multiple testing we used a Bonferroni correction limited to the groups of tests for one phenotype in a population. Calculations were carried out with the SAS software (version 9.1.3). Haplotype frequencies were estimated with EM algorithm and common haplotypes (frequency > 0.03) were analyzed with Haploview. Considering the population sample, this study has 90% power to find odds ratio as small as 1.30.

## Results

**Figure 1:**
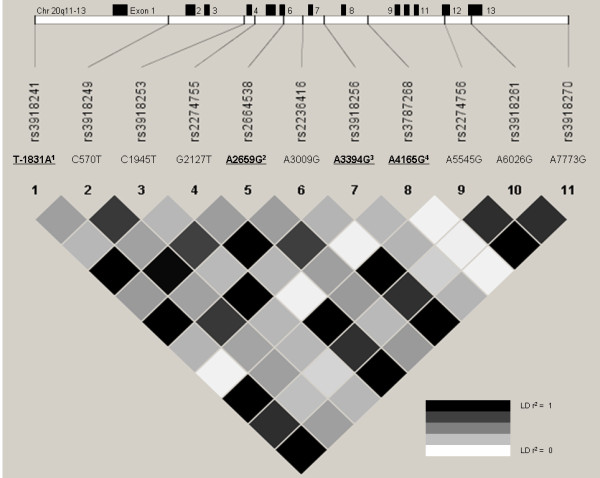
***MMP-9 *gene structure indicating all 13 exons, the position of frequent SNPs (MAF > 0.1) and linkage disequilibrium (r^2 ^plot) based on HapMap data (CEPH population, n = 90)**. ^1) ^SNP rs3918241 is the tagging SNP (R^2 ^> 0.8) for rs2274755, rs2236416, rs2274756, rs3918261 and rs3818270. ^2) ^rs2664538 is the tagging SNP for rs3918249. ^3) ^rs3918256 is the tagging SNP for C1945T. ^4) ^rs3787268 was not in LD with other polymorphisms. Positions based on NCBI sequence database, accession number AL162458.

Using HapMap data, all 11 SNPs in the *MMP-9 *gene could be allocated to four blocks of correlated SNPs showing high levels of linkage disequilibrium (r^2 ^> 0.8) with each other (figure 1) as described in the methods section. One tagging SNP per LD block was selected for genotyping in a cross sectional study population of German children from Munich, Dresden and Leipzig (n = 4,264). Genotyping success rates (call rates) ranged from 91.7% to 96.0% and no significant deviation from Hardy-Weinberg equilibrium was observed (table 1).

First, associations of the four tagging SNPs with main phenotypes asthma and current wheezing were assessed (table 2). SNP rs2664538 showed a trend for an increased risk to develop current wheezing. When analyses were performed for atopic and non-atopic wheezing, association effects were identified especially for non-atopic wheeze. In children homozygous for the polymorphic allele at rs2664538, odds ratios (OR) for non-atopic wheezing was 2.12 (95%CI 1.40-3.21, p < 0.001). Additionally, SNP rs2664538 showed a significant association with non-atopic asthma (OR 1.66, 95%CI 1.12-2.46, p = 0.011). SNPs rs3918241, rs3918256 and rs3787268 showed also an increased risk for the carriers of the homozygous genotype in non-atopic wheeze or non-atopic asthma. When specific IgE of more than 0.35 IU/ml was used to define atopy instead of prick test results, very similar results were observed. In homozygous for the polymorphic allele at rs2664538, odds ratios (OR) for non-atopic wheezing and non-atopic asthma would be 1.92 (95%CI 1.20-3.07, p = 0.006) and 1.50 (95%CI 0.95-2.38, p = 0.083), respectively.

**Table 2:** Odds ratio (and 95% confidence intervals) for associations with asthma and current wheezing in the ISAAC population sample and the effect modification in the strata for atopy (N = 4,264)

Tagging SNPs	Asthma (AS) Current wheeze (CW)	Atopic asthma (AA) Atopic wheeze (AW)	Non-atopic asthma (NA) Non-atopic wheeze (NW)
	**AS **N = **350/3835** **CW **N = **317/3776**	**AA **N = **164/2830** **AW **N = **162/2800**	**NA **N = **167/2830** **NW **N = **138/2800**

**rs3918241** TT/TA/AA N = 2856/964/88	AS 1.10 (0.53 - 2.29) p = 0.806 CW 1.09 (0.50 - 2.38) p = 0.832	AA 0.61 (0.15 - 2.52) p = 0.488 AW 0.94 (0.29 - 3.05) p = 0.920	NA 1.80 (0.76 - 4.25) p = 0.173 NW 1.53 (0.54-4.28) p = 0.418

rs2664538 (Q279R) * AA/AG/GG N = 1655/1821/559	AS 1.27 (0.94 - 1.72) p = 0.121 CW 1.33 (0.97 - 1.82) p = 0.077	AA 0.92 (0.57 - 1.50) p = 0.749 AW 0.81 (0.48 - 1.35) p = 0.415	**NA 1.66 (1.12 - 2.46)** p = 0.011* NW 2.12 (1.40 - 3.20) p < 0.001*

**rs3918256** AA/AG/GG N = 1278/2008/806	AS 1.20 (0.92 - 1.57) p = 0.175 CW 1.17 (0.88 - 1.55) p = 0.285	AA 1.08 (0.72 - 1.61) p = 0.706 AW 0.93 (0.61 - 1.42) 0.750	NA 1.42 (0.98 - 2.05) p = 0.061 **NW 1.60 (1.08 - 2.38)** **p = 0.018**

rs3787268 * GG/GA/AA N = 2419/1310/187	AS 0.97 (0.56 - 1.66) p = 0.906 CW 1.01 (0.58 - 1.77) p = 0.968	AA 0.27 (0.07 - 1.11) p = 0.052 **AW 0.14 (0.02 - 0.99)** **p = 0.021**	NA 1.50 (0.79 - 2.83) p = 0.213 **NW 2.18 (1.17 - 4.06)** p = 0.012*

* Nominal significant differences (p < 0.05) are printed in bold letters, asterisks indicate significance after correction for multiple testing; number of tests performed for one phenotype in a population: four.

When the same 4 SNPs were analyzed separately in the Dresden, Munich or Leipzig population, associations with non-atopic asthma were strongest in Dresden and similar trends for non-atopic asthma were also found in the other two populations for SNP rs2664538. In contrast, a significant protective effect with atopic wheeze was observed for the minor allele of rs3787268 in Leipzig.

Also, lung function parameters had been recorded in a random sample of approximately half of the study population from Munich and Dresden and thus, the effects of *MMP-9 *SNPs on lung function at age 9-11 could be analyzed (table 3).

**Table 3:** Lung function parameters per genotype for non-atopic children with lung function available (population sub-sample from Munich and Dresden)

rs3918241	FEV1% (Mean ± SD)	MEF25% (Mean ± SD)	MEF50% (Mean ± SD)	MEF75% (Mean ± SD)	MMEF % (Mean ± SD)	FVC % (Mean ± SD)	*N*
TT	100.17 ± 10.12	***99.08 ± 30.67***	*99.33 ± 21.57*	100.21 ± 18.57	*99.44 ± 22.16*	100.30 ± 10.38	771

TA	100.15 ± 10.22	***100.15 ± 31.74***	*100.33 ± 23.29*	99.81 ± 19.07	*99.26 ± 21.89*	100.61 ± 11.05	247

AA*	98.18 ± 10.11	***80.64 ± 29.50***	*89.85 ± 24.46*	99.08 ± 21.07	*88.82 ± 24.57*	100.29 ± 9.17	14

p-value (t-test) *	0.468	0.100**	0.101	0.837	0.087	0.976	

**rs2664538**	**FEV1%** **(Mean ± SD)**	**MEF25%** **(Mean ± SD)**	**MEF50%** **(Mean ± SD)**	**MEF75%** **(Mean ± SD)**	**MMEF %** **(Mean ± SD)**	**FVC %** **(Mean ± SD)**	***N***

AA	99.63 ± 10.58	97.73 ± 31.47	98.67 ± 21.18	99.12 ± 18.76	98.55 ± 22.70	99.82 ± 10.76	445

AG	100.35 ± 9.70	100.71 ± 30.15	100.03 ± 21.52	100.45 ± 18.42	99.58 ± 21.24	100.25 ± 10.31	499

GG	99.82 ± 10.60	100.04 ± 32.92	100.11 ± 25.31	102.29 ± 19.88	101.27 ± 23.54	100.47 ± 11.40	148

p-value (t-test) *	0.832	0.792	0.709	0.138	0.297	0.655	

**rs3918256**	**FEV1%** **(Mean ± SD)**	**MEF25%** **(Mean ± SD)**	**MEF50%** **(Mean ± SD)**	**MEF75%** **(Mean ± SD)**	**MMEF %** **(Mean ± SD)**	**FVC %** **(Mean ± SD)**	***N***

AA	99.91 ± 10.75	99.40 ± 31.79	99.21 ± 21.61	**99.43 ± 19.22**	99.72 ± 23.65	99.92 ± 10.66	354

AG	100.07 ± 9.71	99.87 ± 29.97	99.32 ± 21.32	**99.93 ± 18.14**	99.00 ± 20.55	100.05 ± 10.40	555

GG	100.03 ± 10.46	99.51 ± 32.84	100.79 ± 24.33	**102.91 ± 19.85**	100.98 ± 23.50	100.58 ± 11.08	206

p-value (t-test) *	0.983	0.941	0.371	0.116**	0.353	0.478	

**rs3787268**	**FEV1%** **(Mean ± SD)**	**MEF25%** **(Mean ± SD)**	**MEF50%** **(Mean ± SD)**	**MEF75%** **(Mean ± SD)**	**MMEF %** **(Mean ± SD)**	**FVC %** **(Mean ± SD)**	***N***

GG	99.85 ± 10.34	98.32 ± 31.60	99.00 ± 22.14	99.26 ± 19.17	98.67 ± 22.68	100.12 ± 10.67	637

GA	100.31 ± 9.35	99.64 ± 28.68	100.02 ± 20.53	101.47 ± 17.29	99.71 ± 20.97	100.55 ± 9.84	341

AA	101.98 ± 11.61	105.31 ± 36.01	100.09 ± 28.76	101.71 ± 21.39	103.50 ± 23.16	101.91 ± 12.33	58

p-value (t-test) *	0.150	0.125	0.806	0.507	0.170	0.249	

* t test using a recessive model, crude p values showing significant association (p < 0.05) are given in bold; but the effects do not remain significant after correction for multiple testing; FEV1: forced expiratory volume in the first second; MEF: maximum expiratory flows (MEF) at 25, 50 and 75% of vital capacity; MMEF (maximum mid-expiratory flow) is the average expiratory flow over the middle half of the FVC; FVC: forced vital capacity. ** Corrected p valued. Number of tests performed for one phenotype in a population: four.

In non-atopic children, homozygote polymorphic individuals for SNP rs3918241 showed a trend for lower expiratory flows (MMEF% and MEF 25%). However, these effects do not remain significant after correction for multiple testing, and no effect was observed for other lung function measurements such as FEV1, FVC or BHR (data not shown).

SNPs rs2664538, rs3918256 and rs3787268 did not influence lung function parameters significantly. No significant effects on the development of atopy or total serum IgE levels were observed. Haplotype analyses did not contribute additional information to the analysis not observed in the single gene analysis and thus, data are not shown here.

## Discussion

Our systematic study of *MMP-9 *variations suggests that polymorphisms in the *MMP-9 *gene have significant effects on non-atopic forms of asthma. The very specific effects of genetic variations in the *MMP-9 *gene may help to dissect different forms of asthma and elucidate the diversity in the mechanisms leading to airway diseases such as non-atopic asthma and other common, non-atopic forms of childhood wheezing.

**Figure 2:**
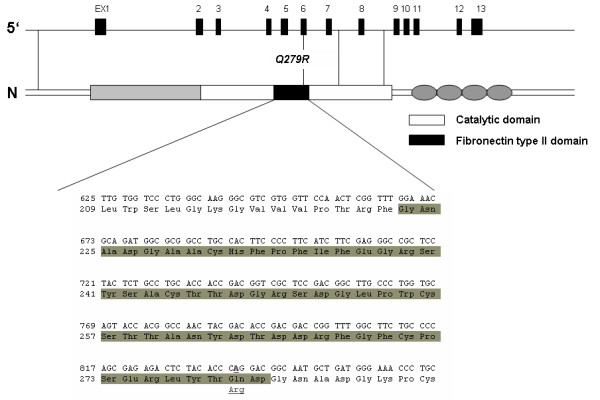
**Localization of coding SNPs on a model structure of MMP-9**. The studied MMP-9 variations rs3918241 (Q279R), is shown in context of MMP-9 structure. Q279 is located in the fibronectin type II domain.

Based on the location of promoter SNP rs3918241, a cis regulatory effect on *MMP-9 *may be suggested. Polymorphism rs3918241 is located exactly in the core sequence of a GATA motif (GTAAAGGAA***G[T/A]TA***ATTATCTC), which may be capable to bind GATA factors, master regulatory transcription factors for the differentiation and perpetuation of human Th2 cells. The variation rs2664538 (Q279R) is located in the MMP-9 fibronectin II domain (figure 2), which presumably enhances the binding of MMP-9 to its substrate, the extra-cellular matrix (ECM) [30]. Using FASTSNP software [31] to predict functional effects of the amino acid change from glutamine to arginine induced by rs2664538, this gene variation seems highly likely to change protein structure and exonic splicing.

Previous *in vivo *studies had also shown that rs2664538 is associated with lower MMP-9 levels [19] and decreased substrate binding [30]. Consistent with our findings, lower MMP-9 levels in sputum have also been associated with airway wall thickening [7] and airflow obstruction. MMP-9 down regulation leads to airway wall thickening, presumably by insufficient degradation and clearance of extra-cellular matrix. In the airways of adult patients with asthma, ECM deposition is not only present in the basal membrane but also around smooth muscle cells and in the adventitial layer, which may gather volume and contribute significantly to the airway wall thickening [32]. By decreasing MMP-9 activity, rs2664538 may directly affect these mechanisms by a decrease in ECM clearance and the subsequent increase in airway wall thickening.

**Figure 3:**
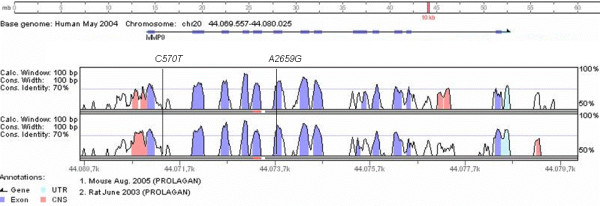
**Comparison of MMP-9 human sequence with mouse and rat sequences**. C570T is located in a non-conserved region, while A2659G (Q279R) is located in the high conserved exon 6.

While the evidence for a direct biological effect of SNP rs2664538 is rather convincing, it may still be argued that rs2664538 may only be a proxy for another SNP within the MMP-9 gene or a neighboring gene, which is responsible and causal for the observed effects. Indeed, rs2664538 is in strong LD (r^2 ^= 1.00) with C570T, a further SNP in intron 1 of the MMP-9 gene. However, by performing extensive *in silico *analysis of the region and the C570T SNP itself using phylogenetic comparisons and transcription factor bind prediction as described in the online supplement, no evidence for a functional role in regulation or transcription factor binding could be allocated to the intron 1 region or the polymorphism (figure 3).

Promoter SNP rs3918241, which was associated with lung function changes in non-atopic children, is in LD with other *MMP-9 *coding variants with putative function. Of the 5 SNPs in LD with rs3918241, two are located in intronic and non-conserved regions, two are exonic variations leading to amino acid changes and one is located in the 3' flanking region. SNPs rs2274756 (P574R) and rs3918261 (R668Q) are located in the hemopexin domain, which may down-regulate the bioavailability of active MMP-9. Furthermore, the interactions with receptors are proposed to be the original function of hemopexin domains in MMPs. Considering LD, this study cannot discriminate which of the linked polymorphisms is responsible for the effect observed between the tagging SNP and lung function parameters.

LD assessments between MMP-9 and neighboring genes did not reveal a significant LD pattern in the region harboring MMP-9 on chromosome 20[23] (data not shown). Thus, it seems very likely that rs2664538 could indeed be responsible for some of the observed effects, potentially due to its direct influences on MMP-9 activity as previously suggested.

It is not obvious why genetic variants in the *MMP-9 *gene are almost exclusively associated with non-atopic forms of wheezing. Without analyzing atopic as well as non-atopic forms of asthma or wheeze, the effects would not have been detected in our population. Thus, it is not surprising that a previous study was unable to identify an effect of *MMP-9 *variations in unstratified asthma[33], as in that case-control study atopic and non-atopic asthma were not analyzed separately. Also not surprisingly, genome wide association studies on asthma had missed the signal; potentially due to the very same reason or to the fact that the SNP effects are not strong enough for GWAS significance levels [34].

The nature of non-atopic wheeze in school children remains unclear. In particular, the etiological factors of such disease and differences of its characteristics with atopic asthma need confirmation. An understanding of whether different genetic variations influence atopic and non-atopic wheeze in later childhood could also provide fresh insight into the mechanisms underlying childhood wheezing. Kurukulaaratchy et al showed that non-atopic wheezing is as frequent as atopic wheezing in an unselected cohort of 10 year old British children[35]. BHR and being diagnosed as asthmatic were significantly more frequent in children with atopic asthma. Characterization of risk factor profiles for these two states revealed different patterns. Heredity in the form of maternal asthma also had a significant effect on the presence of non-atopic wheeze [36]. Clough et al showed that atopic school children with wheeze had lower FEV1 and greater BHR than non-atopic children [37]. All these findings, as well as our results showed that atopic and non-atopic wheezing seem to be different disorders with similar clinical presentation.

It may be hypothesized that different effects on atopic and non-atopic forms of wheezing may be due to the in part contrasting effects of MMP-9 in inflammation and remodeling. While ECM degradation protects from airway remodeling, degradation of ECM also facilitates the influx of inflammatory cells to the airways in allergic inflammation. On the other hand, MMP-9 (and MMP-2) have previously been shown to be essential factors in the clearance of lung inflammatory cells from the airways [15,18]. Considering these data and our findings, one may hypothesize that lower levels of MMP-9 are associated with decreased influx of eosinophils in atopic inflammation, outbalancing the increase in airway remodeling also due to lower MMP-9 levels and neutralizing the effect on a population level. However, this cannot explain why *MMP-9 *is acting differently in atopic and non-atopic inflammation and why the negative effect of increased remodeling due to changes in a matrix metalloproteinase becomes more important in the non-atopic asthma.

## Conclusions

These results suggest that different wheezing disorders in childhood are affected differently by genetic alterations in the *MMP9 *gene and show the need to better study the role of metalloproteinases in airway inflammation and wheezing.

## List of Abbreviations

EM: expectation-maximisation algorithm; ISAAC: International Study of Asthma and Allergy in Childhood; LD: linkage disequilibrium; MAF: minor allele frequency; MALDI-TOF: matrix-assisted laser desorption/ionization time-of flight; SPT: skin prick test; UTR: untranslated region; SNP: single nucleotide polymorphism; CNS: conserved non-coding sequences; HWE: Hardy-Weinberg Equilibrium; BAL: bronchoalveolar lavage; MMP: matrix metalloproteinase; ECM: extra-cellular matrix; FEV1: forced expiratory volume in one second; MEF: maximum expiratory flows; MMEF: maximum mid-expiratory flow: the average expiratory flow; FVC: forced vital capacity.

## Competing interests

The authors declare that they have no competing interests.

## Authors' contributions

LAP developed the study design, performed genotyping, data analysis and drafted the first version of the manuscript; MD participated in data analysis and manuscript preparation; NK and TI participated in genotyping; CV was involved in data collection; EVM contributed to the collection of data; MK supervised all experiments, participated in the collection of data and data analysis and wrote the final version of the manuscript. All authors have read and approved the manuscript.

## Aknowledgements

This work was funded by the German Ministry of Education and Research (BMBF)/national genome research network (NGFN) research grant NGFN 01GS 0429. L. A. Pinto has been also supported by the German academic exchange service (DAAD).
